# Three-dimensional mapping of pipelines using laser ranging and a gyroscope

**DOI:** 10.1038/s41598-023-47856-5

**Published:** 2023-11-21

**Authors:** Songtao Cui, Jie Liang

**Affiliations:** School of Mechanical and Power Engineering, Zhengzhou University, Zhengzhou, 450001 China

**Keywords:** Mechanical engineering, Electrical and electronic engineering

## Abstract

Conventional underground gas pipeline equipment can only map straight pipelines at close distances. As such, a dual-robot mapping technique is proposed in this study, based on laser ranging and a gyroscope, for application to straight, bent, or sloped underground pipelines. This process is facilitated by two robots (a detecting vehicle and a mapping vehicle) alternately moving from the entrance to the exit of a pipe system. The two vehicles acquire data using individual distance sensors and a gyroscope, producing 3D maps of each section. Mapping results can then be produced for the entire pipeline by splicing images from multiple sections. Simulated validation experiments were conducted using horizontal straight, bent horizontal fixed turning radius, sloped uphill or downhill, and composite sections. Results showed this approach could effectively map different types of pipelines with inner diameters of 300–500 mm. Distance errors over 2000 m of travel were within 1 m, while the angular error was within 1.5°, demonstrating our approach to be highly accurate for complex pipeline system mapping.

## Introduction

Urban construction has increased rapidly in recent years and the scale of gas supply pipelines has expanded as a result^1–3^. As such, mapping underground pipeline networks is becoming an indispensable component of the construction process, with an accuracy that is crucial to the future operation, maintenance, renovation, and expansion of municipal gas projects^4–6^. Conventional pipeline mapping often involves a type of passive inertial navigation-based walking robot^7,8^, capable of mapping only straight pipelines over distances of less than 1 km because of accumulated errors, though variations have been proposed in recent years. For example, Sadeghioon et al.^9–12^ mapped pipelines by detecting and tracking leaked magnetic signals, an approach that was accurate for shallow buried pipelines but not applicable to deeper pipelines. Liu et al.^13,14^ used a mapping method based on the spatial curvature of a fiber grating sensor to calculate motion parameters for subsequent measurement points, iteratively mapping the entire pipeline. However, this method suffers from accumulation errors as parameters are calculated using previous points, making it unsuitable for long-distance pipeline mapping. Abbasi et al.^15–20^ developed a pipeline robot that utilized low-frequency electromagnetic waves. This approach offers high mapping accuracy but requires the robot to communicate externally in real time, which is impractical for deeply buried or metal pipelines (due to electrostatic shielding). Yang et al.^21^ improved error divergence for pure inertial guidance systems using an inertial measurement unit, an odometer, and an incomplete constrained algorithm, which reduced mapping errors below 0.15% over distances of 310 m. However, this distance is too short for most practical applications. In the present study, a dual robot mapping method is proposed, based on laser ranging and a gyroscope, for mapping underground gas pipelines with internal diameters of 300–500 mm over distances of 2000 m. The results of validation tests are provided for various types of pipelines, including horizontal straight, bent horizontal fixed radius, sloped uphill or downhill, and composite sections.

## Pipeline mapping method

**Figure 1:**
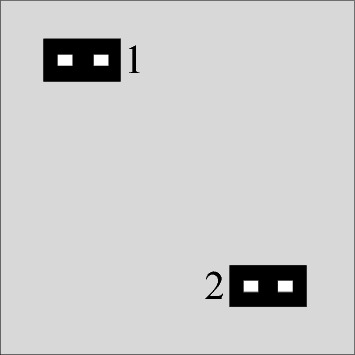
Distance sensor distribution.

**Figure 2:**
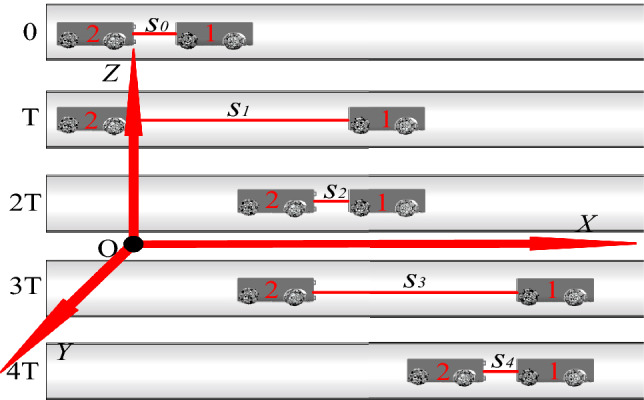
The motion technique.

The proposed technique utilizes a detecting vehicle and a mapping vehicle for underground gas pipeline mapping. A gyroscope in the detecting vehicle measures turning angles in bent pipelines and the slope angle for uphill and downhill sections. Two-point laser ranging distance sensors, shown in Fig. 1, were fixed on the head of the mapping vehicle. These were used to measure distances between the two vehicles, which provided a local coordinate system to determine pipeline section lengths. As shown in Fig. 2, the detecting vehicle (robot 1) moved forward from time 0 to T. The mapping vehicle (robot 2) then moved during another period T, completing one full motion period. This process was repeated until the detecting vehicle identified a bend in the pipeline or an uphill/downhill slope during motion *k*, at which point the robot stops. Individual motion periods are marked and recorded as *m*_1_, *m*_2_, …, *m*_*k*_ for subsequent calculations. The mapping vehicle simultaneously stored data from two distance sensors in each motion period, taking the average distance of *s*_0_, *s*_1_, …, *s*_2k−1_ for subsequent calculations. A 3D coordinate system was then established as shown in Fig. 2, with the pipeline inlet center as the coordinate origin, the forward direction of the two vehicles serving as the X-axis, the vertical direction forming the Z-axis, and the direction perpendicular to the *x*–*z* plane defining the Y-axis. Coordinates for the detecting vehicle can be represented by the location of its tail center.

Motion proceeded as follows: At time *t* = 0, the abscissa of the detecting vehicle is given by *x*_0_ = *s*_0_. During the first motion step *m*_1_, the detecting vehicle begins to move at time *t* = 0. The distance between the two vehicles is given by *s*_1_ at time T and by *s*_2_ at time 2T, while the abscissa of the detecting vehicle is *x*_1_ = *s*_1_ after *m*_1_. In the second motion step *m*_2_, the detecting vehicle begins to move at time 2T. The distance between the two vehicles is then *s*_3_ at time 3T and s_4_ at time 4T, while the abscissa of the detecting vehicle is *x*_2_ = *s*_1_ + *s*_3_ − *s*_2_ after *m*_*2*_. After the third motion step *m*_*3*_, the abscissa of the detecting vehicle is given by *x*_3_ = *s*_1_ + *s*_3_ − *s*_2_ + *s*_5_ − *s*_4_. When the detecting vehicle identifies a bent or sloped pipeline and stops during *m*_*k*_, the abscissa of the detecting vehicle can be represented as:

1 $$ \begin{aligned} x_{k} & = s_{1} + s_{3} - s_{2} + \cdots + s_{2k - 1} - s_{2k - 2} \\ & = \sum\limits_{i = 1}^{k} {s_{2i - 1} - } \sum\limits_{i = 2}^{k} {s_{2i - 2} } . \\ \end{aligned} $$

Assuming the detecting vehicle identifies a bent or sloped pipeline during motion *m*_*j*_, the abscissa of the detecting vehicle can be expressed as:

2 $$ x_{j} = \left\{ {\begin{array}{*{20}l} {s_{1\,} } \hfill & {\quad (j = 1)} \hfill \\ {\sum\limits_{i = 1}^{j} {s_{2i - 1} - } \sum\limits_{i = 2}^{j} {s_{2i - 2} } } \hfill & {\quad (j > 1)} \hfill \\ \end{array} } \right.. $$

In this study, *x*_j_ was assumed to be the abscissa of the starting point for a bent or sloped pipeline section (the turning angle and slope were detected by the gyroscope). Equation (2) can then be used for subsequent 3D mapping of horizontal straight, bent horizontal fixed turning radius, sloped uphill or downhill, and composite pipeline sections.

### Mapping of horizontal straight pipeline sections

When the detecting vehicle identifies a bent or sloped pipeline during motion step *m*_*j*_, the length of the straight pipeline *L* is assumed to be equal to the abscissa *x*_j_:

3 $$ L = x_{j} = \left\{ {\begin{array}{*{20}l} {s_{1\,} } \hfill & {\quad (j = 1)} \hfill \\ {\sum\limits_{i = 1}^{j} {s_{2i - 1} - } \sum\limits_{i = 2}^{j} {s_{2i - 2} } } \hfill & {\quad (j > 1)} \hfill \\ \end{array} } \right.. $$

**Figure 3:**
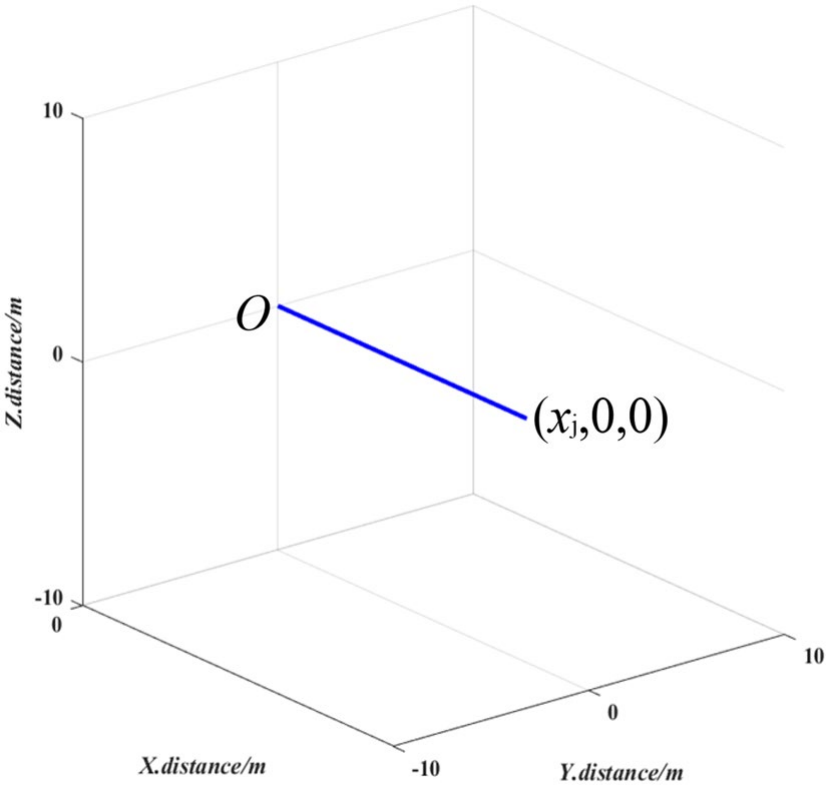
3D mapping results for a horizontal straight pipeline section.

As shown in Fig. 3, 3D mapping results for a horizontal straight pipeline consist of a line segment from the original point to the point (*x*_j_,0,0).

### Mapping of bent horizontal fixed radius pipeline sections

**Figure 4:**
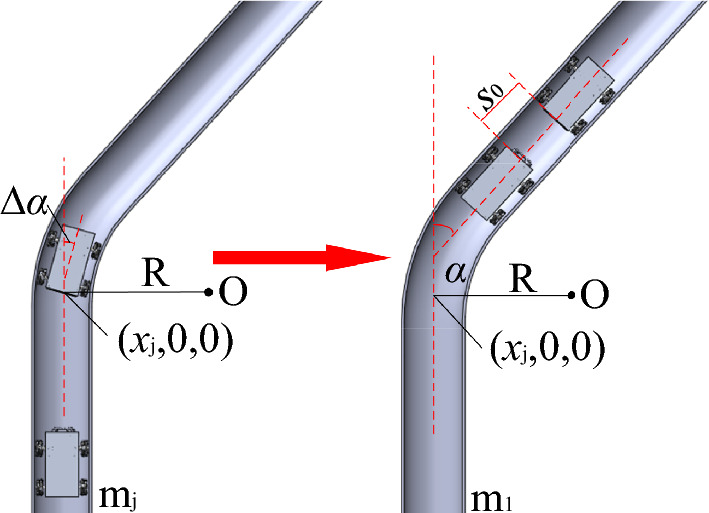
The mapping technique for horizontal fixed turning radius bent pipeline sections.

**Figure 5:**
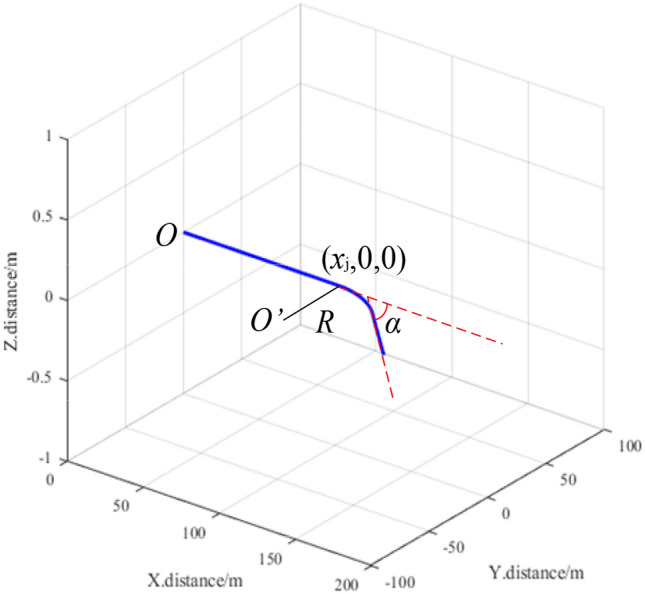
3D mapping results for a horizontal fixed turning radius bent pipeline section.

Bent pipeline sections were assumed to exhibit a fixed turning radius R, with an uncertain turning angle *α*. In the process of motion, the horizontal vehicle body angle *α* was measured in real time using a gyroscope module. As shown in Fig. 4, the body angle ∆*α* will exceed a preset threshold *α*_th_ when the detecting vehicle enters a bent pipeline. At this point, the detecting vehicle stops and the abscissa of the entrance is recorded as *x*_j_. A control module is then activated to move the two vehicles until the change in ∆*α* is stable. The robots are assumed to have cleared the bent section when the turning angle *α* returns to a normal range. Sample 3D mapping results are provided for a bent section in Fig. 5. This pipeline extends from the origin to the point (*x*_j_,0,0) and turns with a fixed radius R. After turning through the pipeline, the state of the two robots is reset and a 3D coordinate system is re-established, with the origin O located at the center of the plane where the two distance sensors are fixed. The distance between the two vehicles is then reset to the initial distance *s*_0_ and the next round of alternating forward motion resumes. Individual mapping results are spliced to form a single image after the last motion step.

### Mapping of sloped uphill and downhill pipeline sections

**Figure 6:**
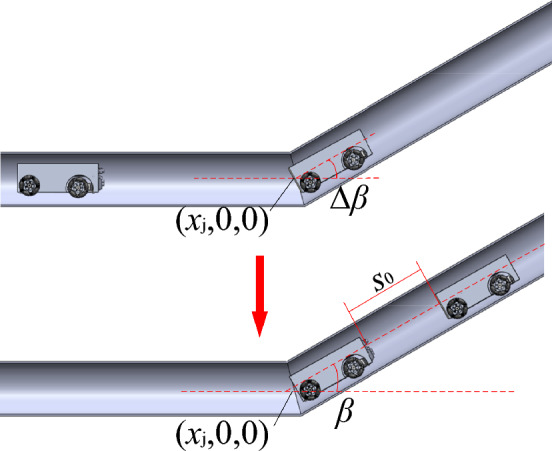
The mapping technique for uphill or downhill pipeline sections.

**Figure 7:**
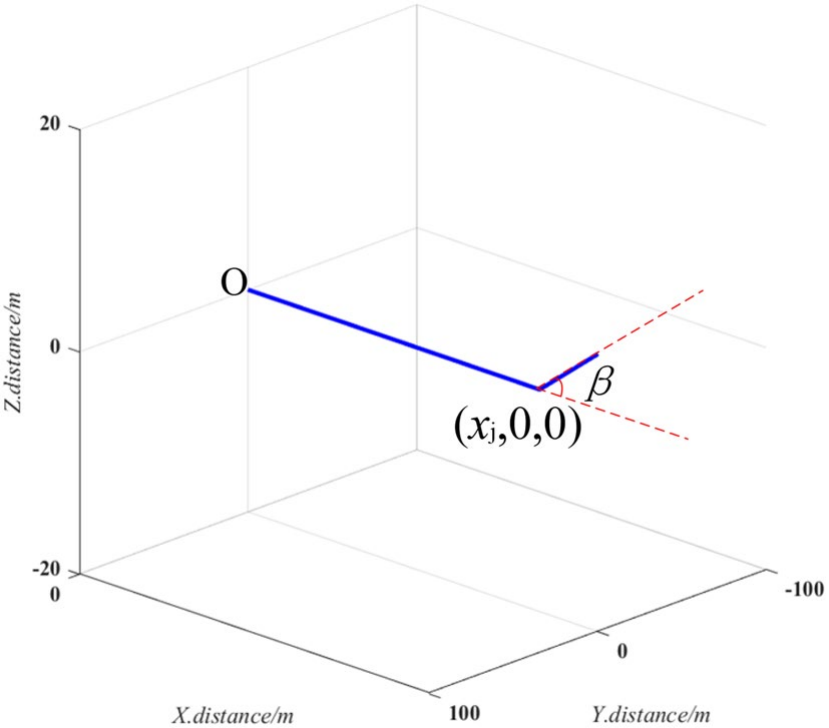
3D mapping results for an uphill pipeline.

The detecting vehicle assumes it has reached a sloped uphill or downhill section when the vertical body slope ∆*β* exceeds a preset threshold *β*_th_. The vehicle then stops at the beginning of this section and records the abscissa *x*_j_, as shown in Fig. 6. A control module is then activated to guide the two vehicles forward until changes in ∆*β* are stable. At this point, the two robots are assumed to have cleared the sloped pipeline section when the slope *β* returns to a normal range. Sample 3D mapping results are shown in Fig. 7, where the pipeline extends from the origin to the point (*x*_j_,0,0) and then ascends with a slope of *β*. After exiting the pipeline, the state of the vehicles and the 3D coordinate system are both reset as described above.

### Solution process

**Figure 8:**
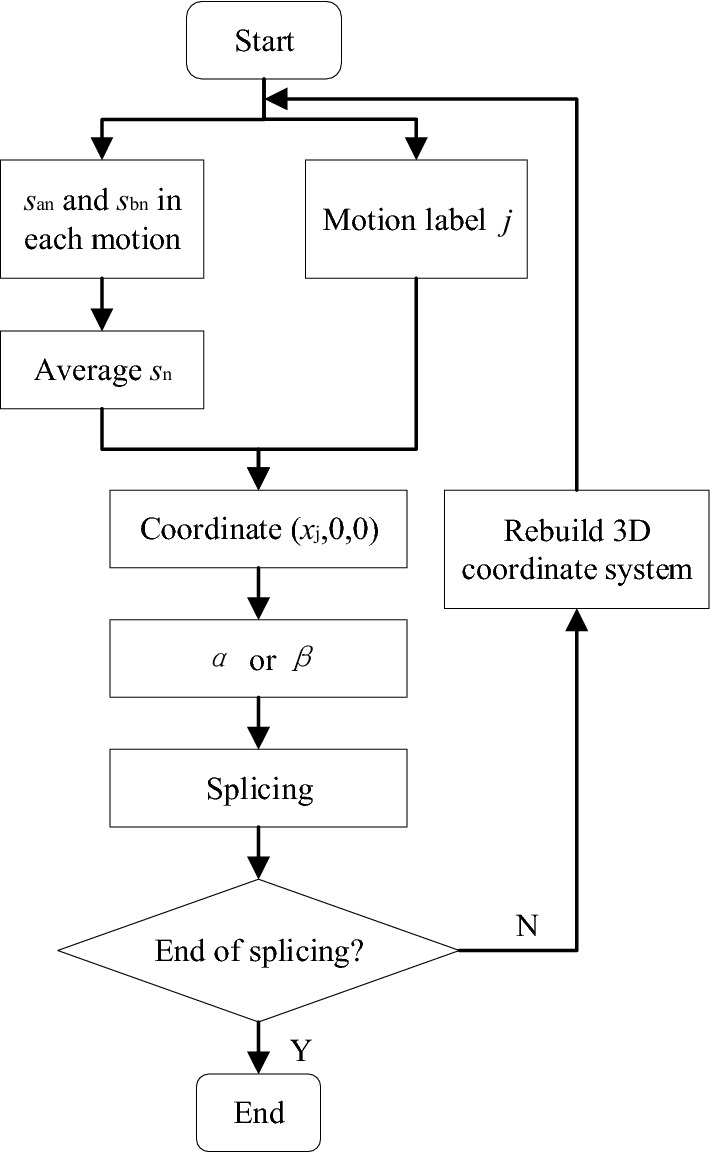
The solution process.

A full 3D mapping result can be acquired using the measured data *s*_a_, *s*_b_, m_j_, *α*, and *β*, once the two robots have exited the pipeline (see Fig. 8). A map is generated whenever the robots encounter a bent or sloped pipeline, with the center point of the entrance serving as the coordinate origin. The distance *s*_n_ between the two robots is acquired by averaging *s*_a_ and *s*_b_ for the two sensors. The coordinates (*x*_j_,*y*_j_,*z*_j_) for the starting/ending point of each section are denoted by *α* and *β*. Finally, 3D mapping results for the entire pipeline were acquired by splicing.

## Robot composition

### Mechanical structure of detecting and mapping vehicles

**Figure 9:**
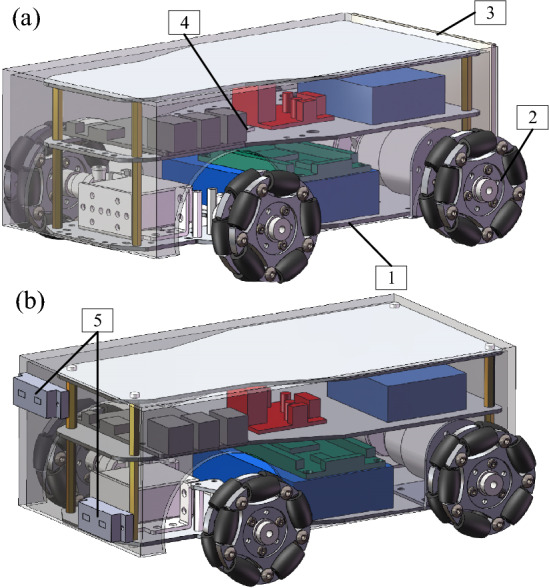
3D models of the (**a**) detecting and (**b**) mapping vehicles. Labels include: 1—Ackerman chassis, 2—omnidirectional wheels, 3—reflector, 4—gyroscope, and 5—point laser distance sensor.

The two robot vehicles utilized an Ackman chassis structure for simpler turning and improved mobility in the pipeline. Omnidirectional wheels also provided a larger contact area for increased stability, as shown in Fig. 9. The vehicle body included a two-story board structure to make full use of the available vertical space. A two-point laser distance sensor was then fixed to the head of the mapping vehicle and used to measure distances between the robots, facilitated by a reflector mounted to the rear of the detecting vehicle.

### Detecting and mapping vehicle control systems

**Figure 10:**
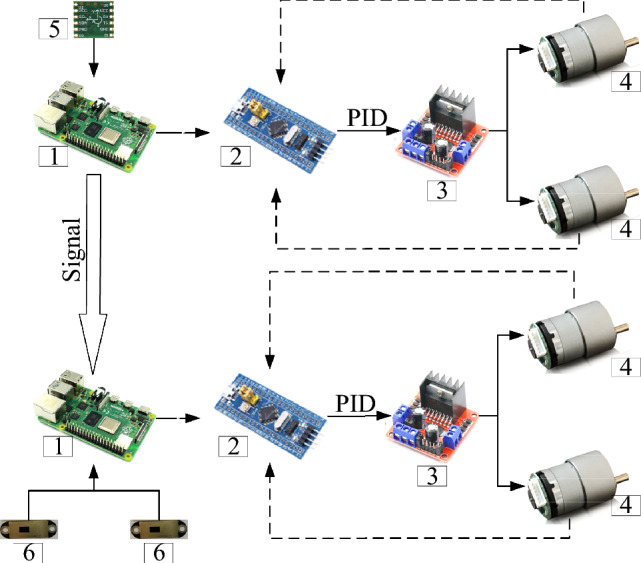
Control system (1—main control module, 2—auxiliary control module, 3—driver module, 4—DC motor, 5—gyroscope, and 6—point laser distance sensor).

Both vehicles exhibited an onboard primary control module (PCM) and an auxiliary control module (ACM). The detecting vehicle use socket to communicate with the mapping vehicle. The detecting vehicle utilized a Raspberry Pi 4b+ development board as the PCM, as shown in Fig. 10. This device provided four essential functions: (1) recording motion section marks (*m*_*j*_), (2) acquiring real-time gyroscope data (accuracy: X/Y axis is 0.2°, Z axis is 1°), (3) controlling the mapping vehicle through socket communication, and (4) sending motion signals to the ACM. The ACM featured an stm32c8t6 development board, used to receive signals from the PCM and control two DC motors via an L298N driver module. Encoders on the two motors provided rotation speed feedback to achieve uniform motion using the incremental PID algorithm. As shown in Fig. 10, the mapping vehicle also included a Raspberry Pi 4b+ development board, which served as the PCM. This device provided three essential functions: (1) recording distance sensor data, (2) collecting signals from the detecting vehicle through socket communication, and (3) sending motion signals to the ACM. When the distance between two vehicles detected by the point laser distance sensor is little than safety distance, the mapping vehicle stops. The safety distance can be set to avoid collisions.

### Turning analysis

**Figure 11:**
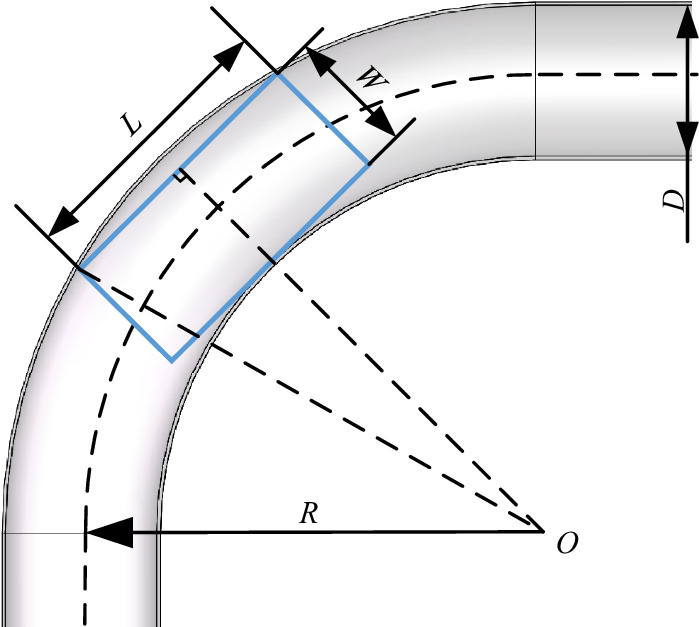
An analysis of robot turning.

**Figure 12:**
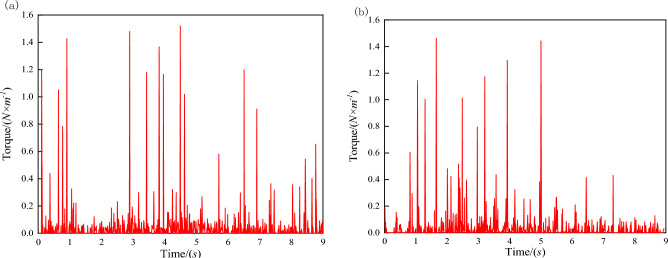
Torque diagrams for (**a**) the internal motor and (**b**) the external motor.

**Figure 13:**
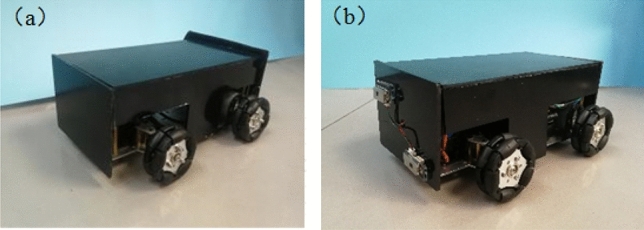
Photographs of the (**a**) detecting and (**b**) mapping vehicles.

**Figure 14:**
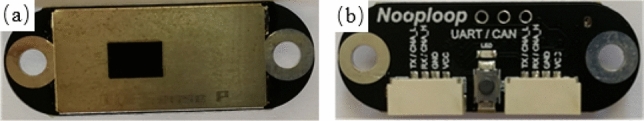
The point laser distance sensor.

Longer and wider vehicle bodies increase the difficulty of moving through bent pipelines, necessitating an analysis of robot dimensions. As shown in Fig. 11, the length *L* and width *W* of the robot should satisfy:

4 $$ L \le L_{\max } = 2\sqrt {(R + 0.5D)^{2} - (R - 0.5D + W)^{2} } , $$

where O denotes the turning center, R is the turning radius, D is the inner diameter of the pipeline, *L* is the length of the vehicle body, and *W* is the width of the vehicle body. In the presented experiments, the inner diameter of the pipeline was 300 mm, the maximum length *L* of the vehicles was 240 mm, the vehicle width *W* was 120 mm, and the minimum pipeline turning radius *R* was 600 mm. Equation (3) suggests the geometric parameters for the two vehicles satisfy this turning requirement. A dynamic analysis of the detecting vehicle was performed and assumed to apply to both vehicles, due to their similar structure. Motion of the detecting vehicle was simulated by Adams at a speed of 1 m/s in a bent pipeline with an inner diameter of 300 mm and a turning angle of 90°. Torque diagrams for the internal and external DC motors are shown in Fig. 12a and b, respectively. These diagrams suggest the two vehicles can pass smoothly as the maximum torque for the two motors does not exceed the rated torque of 1.6 N/m. Photographs of the detecting and mapping vehicles are shown in Fig. 13a and b, respectively, with geometric parameters provided in Table 1. In our setup, the point laser distance sensors (which utilized serial port communication) exhibited a maximum measurement distance of 12 m, with a resolution ratio of 1 cm. The distance for each motion section was set to 5 m. The figure of the point laser distance sensor is shown in Fig. 14a and b.

**Table 1:** Geometric parameters for the detecting and mapping vehicles.

	Length (mm)	Width (mm)	Height (mm)
Detecting vehicle	239	178	84
Mapping vehicle	236	178	84

## Experiment

### Horizontal straight pipeline sections

**Figure 15:**
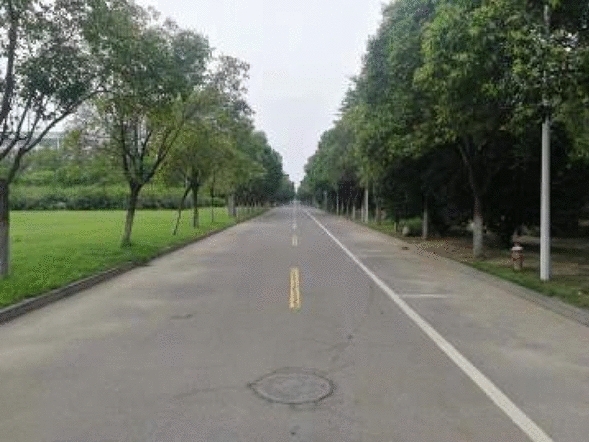
The road used to simulate a straight pipeline.

**Figure 16:**
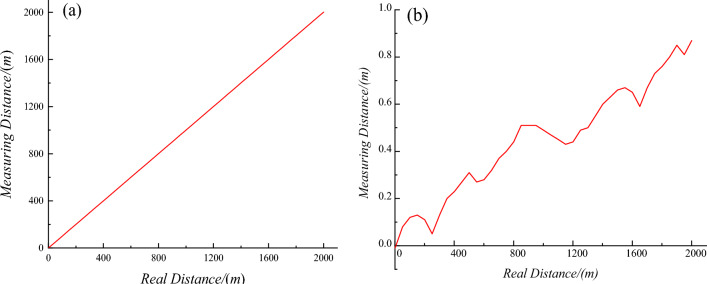
Measurement results for a straight pipeline, including comparisons of (**a**) measured and actual distances and (**b**) measurement errors and actual distances.

As shown in Fig. 15, the viability of this method in horizontal straight pipeline sections was evaluated over a distance of 2000 m on a flat road, used to simulate a pipeline. In this experiment, *s*_0_ was set to 1 m. Sample points were set every 50 m and used to compare the experimental distance with the actual distance measured by a band tape, as shown in Fig. 16a. The resulting measurement errors are provided in Fig. 16b, where the largest error was less than 1 m.

### Horizontal fixed turning radius bent pipeline sections

**Figure 17:**
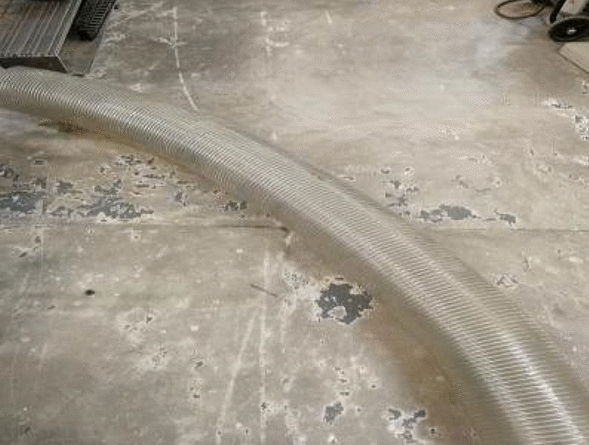
The rubber pipe used to simulate a bent pipeline.

**Figure 18:**
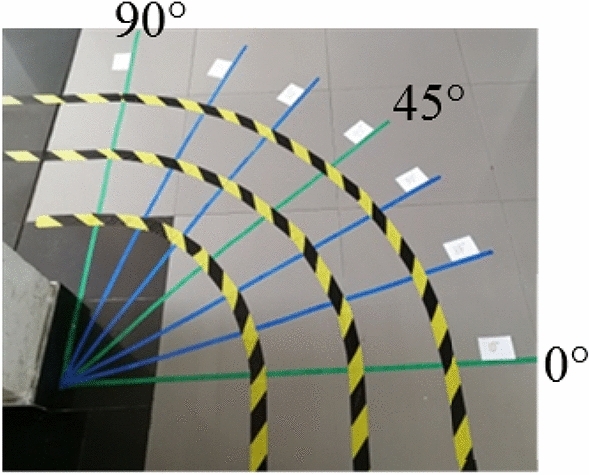
The experimental pipeline track.

**Figure 19:**
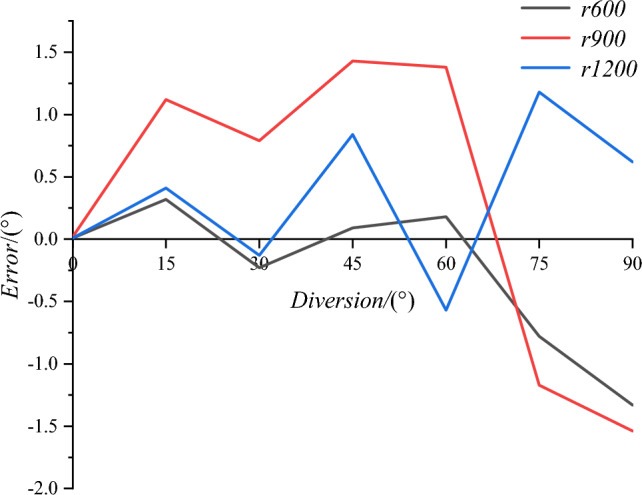
Experimental results for a fixed turning radius bent pipeline.

The feasibility of our technique for fixed turning radius horizontal bent pipelines was evaluated using rubber tubing with an outer diameter of 308 mm and an inner diameter of 300 mm, as shown in Fig. 17. The inner diameter of the tubing was consistent to simulate an actual pipeline. In addition, warning tape was used to construct tracks with turning radii of 600 mm, 900 mm, and 1200 mm, as shown in Fig. 18. Colored tape was included on the track base to mark the actual turning angles of 0°, 15°, 30°, 45°, 60°, 75°, and 90°. The rubber tubing was then positioned on the track to simulate a horizontal bent pipeline with varying turning radii and angles. The largest error between the measured angle and actual turning angle was less than 1.5°, as shown in Fig. 19.

### Sloped uphill and downhill pipeline sections

**Figure 20:**
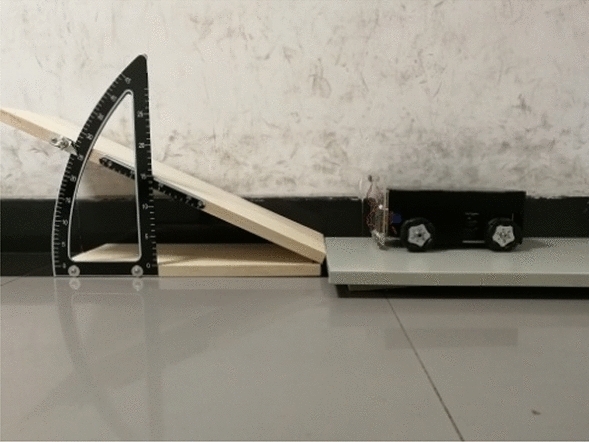
The adjustable slope.

The effectiveness of our method for uphill and downhill pipelines was assessed using an adjustable slope, as shown in Fig. 20. Varying angles were used to compare the measured slope with the actual slope, producing a largest error of less than 1.5°, as shown in Table 2.

**Table 2:** Experimental results for the uphill and downhill pipelines.

Actual slope angle (°)	10	20	30	40
Measured slope angle (°)	9.85	20.77	31.04	40.51
Error (°)	− 0.15	0.77	1.04	0.51

### Composite pipeline sections

**Figure 21:**
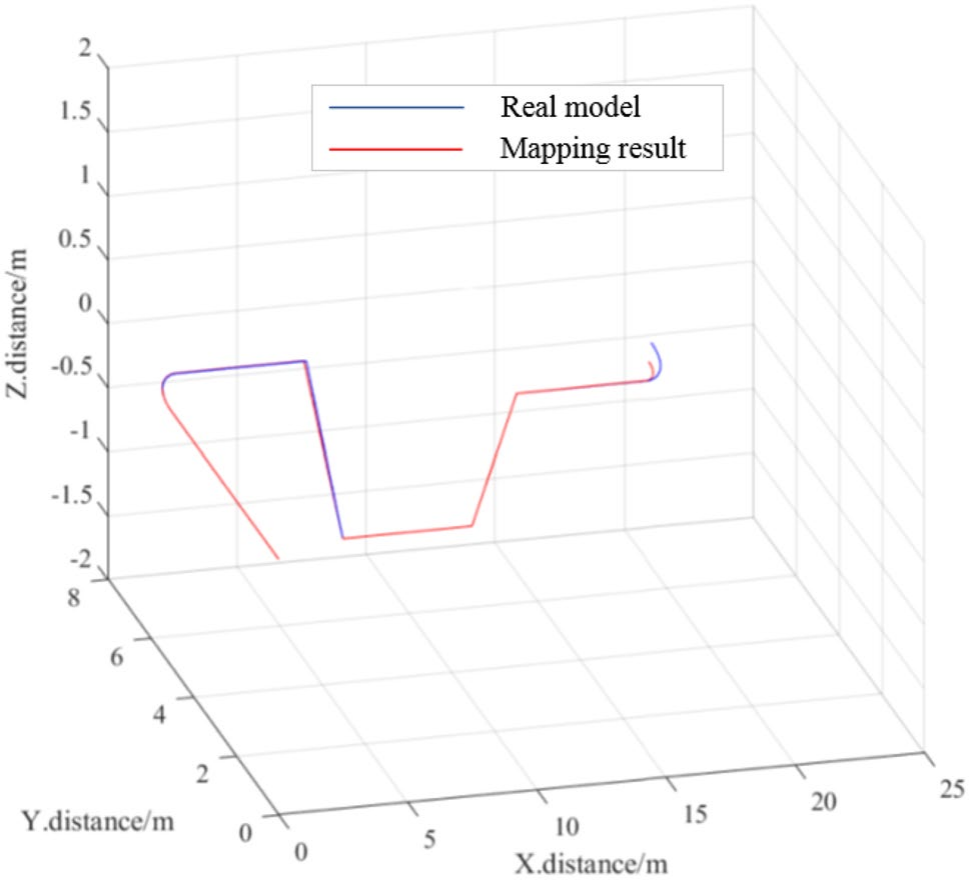
Real model and mapping results for the composite pipeline.

A strictly simulated composite pipeline was established by combining multiple pipeline sections, producing the measured results and actual data shown in Table 3. In this experiment, the largest length error was 0.033 m, the largest turning angle error was 0.9°, and the largest slope angle error was 0.6°. The actual 3D model and mapping results for the composite pipeline are shown in Fig. 21a and b, respectively.

**Table 3:** Experimental results for a composite pipeline.

Number	Type of pipeline section		Length (m)		Slope angle (°)		Turning angle (°)	
	Actual	Measured	Actual	Measured	Actual	Measured	Actual	Measured
1	Straight	Straight	5	5.016				
2	R1200 Bent	R1200 Bent	1.2	1.202			90	90.9
3	Straight	Straight	5	4.997				
4	Downhill	Downhill	1.2	1.210	− 45	− 45.2		
5	Straight	Straight	5	5.033				
6	Uphill	Uphill	1.2	1.198	30	30.6		
7	Straight	Straight	5	4.997				
8	R600 Bent	R600 Bent	1.2	1.207			− 90	− 90.5

## Conclusion

A dual robot technique utilizing laser ranging and a gyroscope was proposed for mapping underground gas pipelines with inner diameters of 300–500 mm. The primary contributions of this study are as follows:

1. Horizontal straight, bent horizontal fixed turning radius, and sloped uphill and downhill pipeline sections were simulated and tested experimentally using the proposed dual-robot pipeline mapping technique.
2. The designs of the detecting and mapping vehicles were optimized for typical pipeline dimensions, using dynamic simulation analysis software (Adams).
3. Validation experiments were conducted to assess the viability of our technique for varying pipeline sections, with length errors of less than 1 m over distances of 2000 m, turning angle errors of less than 1.5°, and slope angle errors of less than 1.5°. The largest length error in the composite pipeline was 0.033 m, while the largest turning angle error was 0.9° and the largest slope angle error was 0.6°.

Unlike conventional mapping methods, this technique could be applicable to the mapping of straight, bent, or sloped underground pipelines. Future work will involve combining this approach with odometer and accelerometer data, fusing multi-modality sensor measurements to achieve a more accurate mapping result. In addition, this technique is not suitable for uphill or downhill pipelines with slope angles exceeding 45°, so new motion mode and mechanical structure of robots are needed for improvement.

## Data Availability

The datasets used and/or analysed during the current study available from the corresponding author on reasonable request.
